# The Application of Polymer Inclusion Membranes Based on CTA with 1-alkylimidazole for the Separation of Zinc(II) and Manganese(II) Ions from Aqueous Solutions

**DOI:** 10.3390/polym11020242

**Published:** 2019-02-01

**Authors:** Elzbieta Radzyminska-Lenarcik, Malgorzata Ulewicz

**Affiliations:** 1 Faculty of Chemical Technology and Engineering, UTP University of Science and Technology, PL85326 Bydgoszcz, Poland; 2 Faculty of Civil Engineering, Czestochowa University of Technology, PL42201 Czestochowa, Poland; ulewicz@bud.pcz.pl

**Keywords:** polymer inclusion membrane, 1-alkylimidazole, zinc(II), manganese(II), AFM, SEM

## Abstract

Polymer cellulose triacetate membranes doped with 1-alkylimidazole as fixed carriers were applied for the investigation of the facilitated transport of Zn(II) and Mn(II) ions from an aqueous sulphate feed phase (c_M_ = 0.001 mol/dm^3^). For the polymer inclusion membranes (PIMs) doped with 1-alkylimidazole (alkyl – from hexyl up to decyl), the following patterns of transport selectivity were found: Zn(II) > Mn(II). The highest initial flux of Zn(II) ions (2.65 µmol/m^2^·s) was found for PIMs doped with 1-decyl-imidazole, whereas the best Zn(II)/Mn(II) selectivity coefficients equal to 19.7 were found for 1-hexyl-imidazole. Permeability coefficients for Zn(II) and Mn(II) ions transported across PIMs increase with an increase in the p*K*_a_ values of 1-alkylimidazole. The polymer membranes of cellulose triacetate-*o*-NPPE with 1-alkylimidazole were characterised by scanning electron microscopy, non-contact atomic force microscopy and thermal analysis techniques. The influence of membrane morphology on the Zn(II) and Mn(II) transport process was discussed.

## 1. Introduction

Nowadays, the necessity to protect the environment and the gradual depletion of metal-bearing mineral resources have caused a certain convergence of interests, which results in the necessity to remove heavy metals from effluents and solid waste; at the same time, metal-bearing waste has become a secondary raw material for the production of economically valuable metals, e.g., zinc. This causes increased interest of entrepreneurs and scientists in seeking successful and cheap methods for recovering valuable metals, including zinc, from secondary raw materials. The global production of refined zinc in 2017 amounted to 13,234 thousand tonnes [1], of which secondary zinc produced from secondary raw materials constituted approx. 30%. The zinc recovery process depends on the form and concentration of this metal in the metal-bearing material and on the degree of contamination of the raw material with other compounds.

Currently, zinc(II) and the ions of other metals can be selectively separated by means of various separation methods, such as solvent extraction [2,3,4], ion flotation [5,6,7,8,9], ionic exchange [10,11], solvent sublation [12,13], sorption [14,15], and liquid membranes [16,17,18,19,20], in which the stages of extraction and re-extraction proceed simultaneously. The separation of zinc ions can be performed by means of extracting agents/commercial carriers, i.e., organophosphorous acids or Cyphos [20,21,22,23], macrocyclic [18,23,24], as well as imidazole derivatives [25,26,27,28].

Waste alkaline zinc-manganese batteries and zinc-carbon batteries are a valuable source of secondary zinc. These waste batteries contain considerable amounts of zinc (5%–21%) and manganese (23%–33%), as well as minor amounts of other metals (0.1%–1.0% Fe, 5%–7% K, approx. 0.01% Ni) [29] and they can be processed in pyrometallurgical processes: Citron, Tera, Waelz, Batric and Sumitomo, as well as hydrometallurgical processes: Batrec (Switzerland), Batenus (Germany), Zincex MZP (Spain) and TNO (the Netherlands, Germany) [30,31,32]. However, new, more economically effective hydrometallurgical methods of recovering this metal are still being sought.

A typical hydrometallurgical metal production process consists of four major technological steps such as leaching, phase separation, the separation of metal ions from aqueous solutions and the precipitation of metals or their compounds from the aqueous phase [29,33,34]. Among the listed technological operations, particular attention should be paid to the process of separating metal ions in an aqueous solution, which decides about the degree of purity of the resulting products. This process can be carried out by means of methods such as liquid–liquid extraction, ion exchange, ion flotation or transport through liquid membranes. The selection of a proper method and conditions for conducting the process decides about its selectivity and the possibility of application on an industrial scale.

Membrane technologies have become an important alternative to conventional processes employed for wastewater treatment, separation and recovery of target metals [35,36]. The selective transport of metal ions has been widely studied with supported (SLMs) and polymer inclusion (PIMs) liquid membranes [16,17,18,19,20,21,22,23,24,25,26,27,28,35,36,37]. Their high selectivity, high diffusion rates and the ability to concentrate ions have made them particularly useful [35,38].

The aim of this work was to check the possibility of separation and recovery of Zn(II) from equimolar sulphate mixtures of Zn(II)-Mn(II) ions from aqueous solutions using PIMs doped with hydrophobic 1-alkyl-imidazoles (alkyl = 1-hexyl, 1-heptyl, 1-octyl, 1-nonyl-, 1-decyl). 

The purpose of work also included investigation of the dependence of the physical properties of CTA-based membranes on their efficiency in the separation of Zn(II)-Mn(II) ions during transport across such membranes.

## 2. Experimental

### 2.1. Reagents and Apparatus

Inorganic chemicals: zinc(II), manganese(II) sulphate and tetramethylammonium hydroxide were of analytical grade and were purchased from POCh (Gliwice, Poland). Aqueous solutions were prepared with double distilled water (conductivity 5 µS/m). 

**Figure 1:**
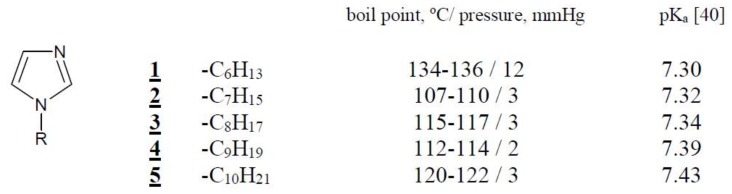
The chemical formula 1-alkyl-imidazoles and their physicochemical properties.

Organic reagents, i.e., cellulose triacetate (CTA), *o*-nitrophenyl pentyl ether (*o-*NPPE), and dichloromethane (all from Fluka, Switzerland) were of analytical grade and were used without further purification. 1-alkylimidazoles **1**–**5** (Figure 1) were synthesized according to the procedure described in paper [39]. The p*K*a value of all carrier is obtained from reference [40].

### 2.2. Polymer Inclusion Membrane Preparation

**Figure 2:**
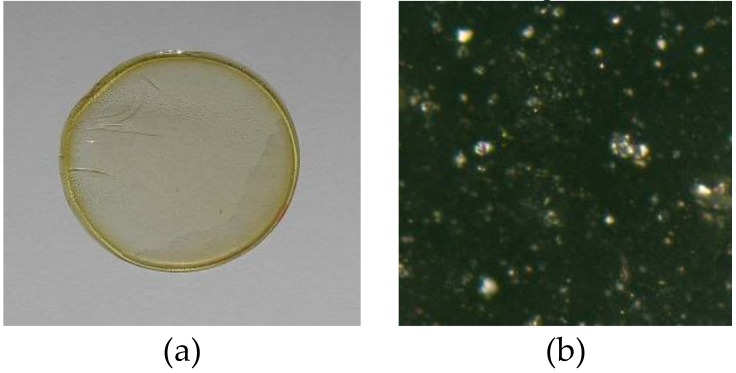
PIM: CTA-*o*-NPPE-1-hexyl-imidazole: before process—real diameter 44 mm (**a**) photo from the otoscope microscope—enlargement 1000 times (**b**).

The membranes were prepared according to the procedure reported in the previous paper [25]. A solution of cellulose triacetate (CTA), plasticizer (*o*-nitrophenyl pentyl ether (*o-*NPPE)), and 1-alkylimidazoles **1**–**5** (Figure 1) as ion carriers in dichloromethane was prepared. 25 cm^3^ of the solution was poured into a membrane mould consisting of a 9.0 cm diameter glass ring fixed on a glass plate with cellulose triacetate-dichloromethane glue. After evaporation of the solvent overnight at room temperature the resulting membrane was peeled off from the glass plate by immersion in cold water. Then the membrane was soaked for 12 h in distilled water to ensure its homogeneity. Two discs were cut out from the same preparation to duplicate transport experiments. The wet membrane contained 2.67 cm^3^ *o*-NPPE /1 g CTA, and 1.0 mol/dm^3^ concentration of 1-alkyl-imidazoles (**1**–**5**) based on plasticizer. The example membrane and its photo taken with an optical microscope (NICON ECLIPSE E40 POL) are shown in Figure 2.

### 2.3. Characteristics of PIMs

The thickness of the PIM samples was measured using a digital micrometer (Panametrics^®^ Magna-Mike^®^ 8500 (San Diego, CA, USA)) with an accuracy of 0.1 µm. The thickness of a membrane was measured 10 times for each case and shown as the average value of these measurements, with the standard deviation below 1%. The thickness of membranes before and after transport was found to be the same. The average PIMs thickness varied in the range of 29–32 μm. Experimental reproducibility was high with standard deviation below 1% of the measured values.

A surface characterization study of the polymer membranes was performed using an Atomic-force MultiMode Scanning Probe Microscope IIIa (AFM) (Digital Instruments Vecco Metrology Group, Santa Barbara, CA, USA) according to the procedure described in other papers [24,25,26,27]. Pores characterization was performed using the AFM image processing program NanoScope v.5.12 (Digital Instruments Vecco Metrology Group, Santa Barbara, CA, USA), which enabled the calculation of two parameters: roughness (*R_q_*) and porosity (ε). The *R_q_* parameter is the standard deviation of the *z* values within the box cursor and is calculated as:

(1) $$R_{q}=\sqrt{\frac{\sum {\left(Z_{i}\right)}^{2}}{n}}$$

where: *z_i_* is the current *z* value, *n*- is the number of points within the box cursors.

Scanning electron microscope (SEM) images of the membranes were obtained with the Hitachi SU3500 SEM/EDS (energy dispersive spectroscopy) microscope operated at 10.0 kV. The membranes were visualized in 10.0 μm × 10.0 μm images (front view central and from a perpendicular cross-section along medium).

The thermal stability of the polymeric inclusion membranes was determined over the range of 20–700 °C under nitrogen, in ceramic crucibles. An empty crucible served as a reference. The apparatus used was a SDT 2960 instrument (TG209F3 Tarsus Netzsch, Selb, Germany) operated at a heating rate of 10 °C/min and a nitrogen flow rate of 100 cm^3^/min. The sample weight was 10 mg throughout.

### 2.4. Transport Studies

Transport experiments were carried out in a permeation cell described in an earlier paper by the same authors [24,25,26,27]. The membrane film (surface area 4.9 cm^2^) was tightly clamped between two cell compartments. Both, the feed and the receiving aqueous phases (45 cm^3^ each) were mechanically stirred at 600 rpm. Metal sulphate were used in the feed phase whereas the receiving phase was deionized water. The PIM transport experiments were carried out at 20 ± 0.2 °C. Small samples of the aqueous receiving phase were taken periodically from the sampling port equipped with a syringe and analysed by atomic absorption spectroscopy (AAS Spectrometer, Solar 939, Unicam, London, UK) to determine zinc(II), and manganese(II) concentrations. The pH of the feed phase (5.6) was kept constant using tetramethylammonium hydroxide.

The kinetics of the transport across the PIMs was described as a first-order process with respect to the metal-ion concentration expressed by Equation (2):

(2) $$\ln\left(\frac{c}{c_{i}}\right)=-kt$$

where *c* is the metal ions concentration in the feed phase at a given time (mol/dm^3^), *c_i_* is the initial metal ions concentration in the feed phase, *k* is the rate constant (s^−1^), and *t* is the time of transport (s). [41].

To calculate the value of *k*, ln(*c*/*c_i_*) versus time was plotted. The rate constant values for two independent transport experiments were averaged and standard deviation was calculated. The correlation between ln(*c*/*c_i_*) and time was linear, which was confirmed by the high correlation coefficient (*R*^2^) ranging from 0.9624 to 0.9995. The permeability coefficient (*P*) was calculated according to Equation (3):

(3) $$P=-\frac{V}{A}k$$

where *V* is the volume of the aqueous feed phase [m^3^], *k* is the rate constant and *A* is an effective area of the membrane [m^2^].

The initial flux (*J_i_*) is equal to:

(4) $$J_{i}=P\cdot c_{i}$$

The selectivity coefficient (*S*) was defined as the ratio of initial fluxes for M_1_ and M_2_ metal ions, respectively:

(5) $$S=J_{i,M1}/J_{i,M2}$$

To describe the efficiency of metal removal from the feed phase, the recovery factor (*RF*) was calculated:

(6) $$RF=\frac{c_{i}-c}{c_{i}}\cdot 100\%$$

The reported values correspond to the average values of three replicates, with the standard deviation within 5%.

## 3. Results and Discussion

### 3.1. The Characteristics of Membranes

**Figure 3:**
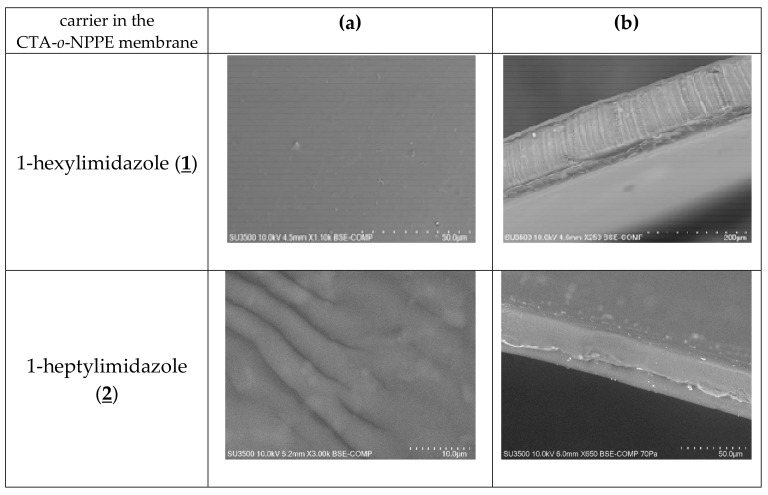

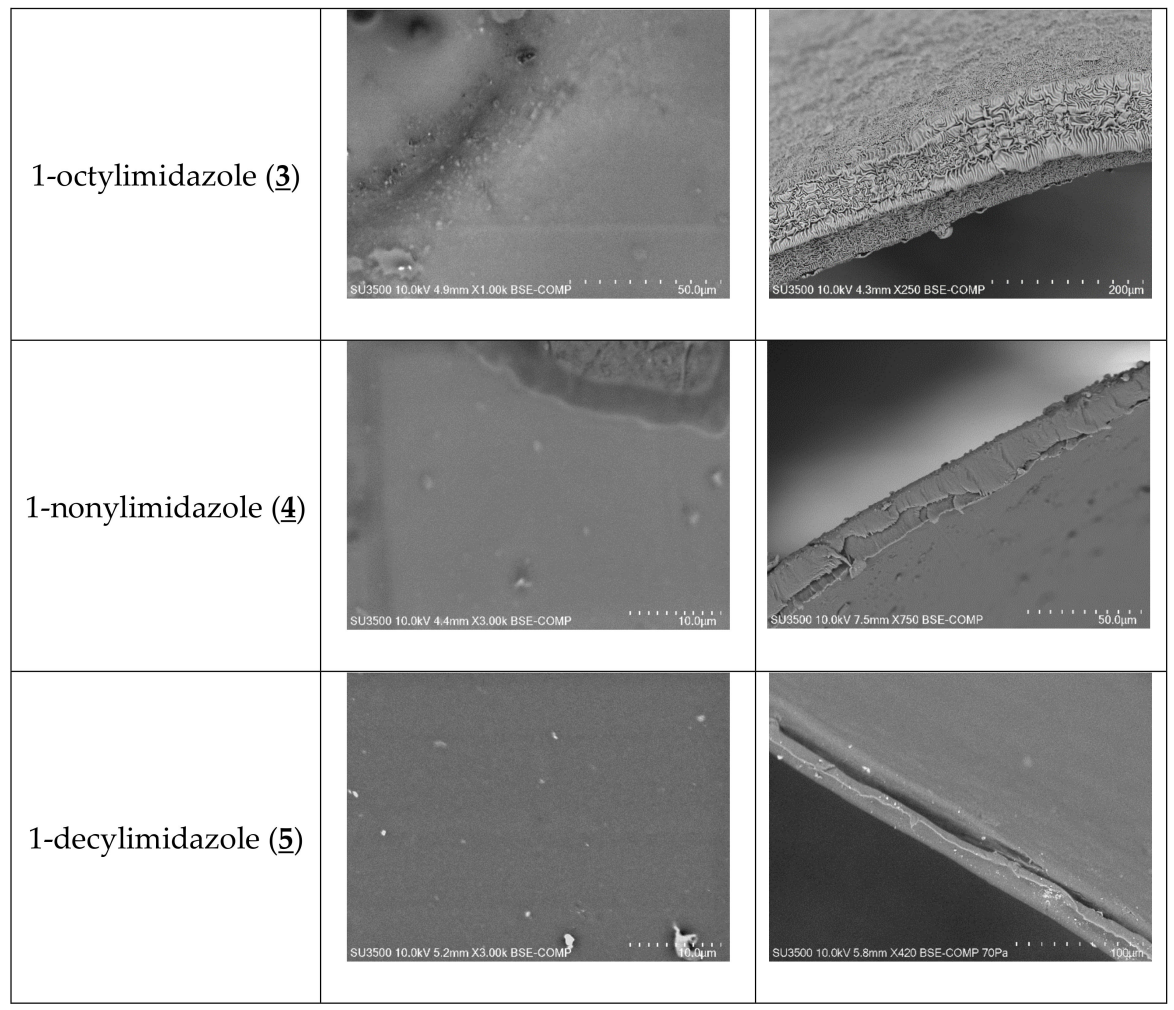
SEM images of PIMs containing 1-alkyl-imidazole as a carrier: front view (**a**), side section (**b**).

The SEM photomicrographs (Figure 3) showed that all membranes had dense and homogeneous structures. Moreover, the images showed visible roughness of film surfaces. Carriers could crystallise in the membrane and, for example, alkylimidazole molecules migrated to the membrane surface, causing its roughness and porosity. 

The distribution of the carrier in the investigated membrane after the evaporation of dichloromethane is homogeneous on the entire surface. PIMs containing 1-alkyl-imidazole are dense and homogenous.

**Figure 4:**
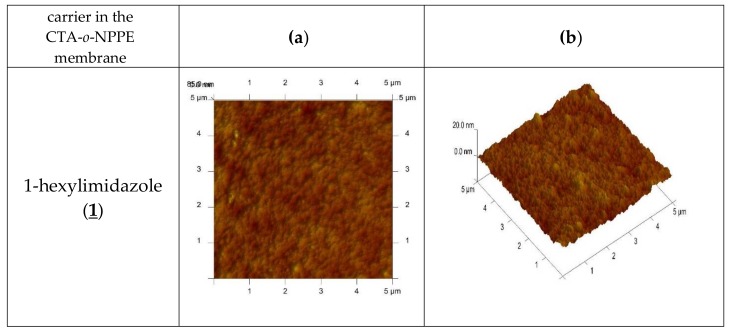

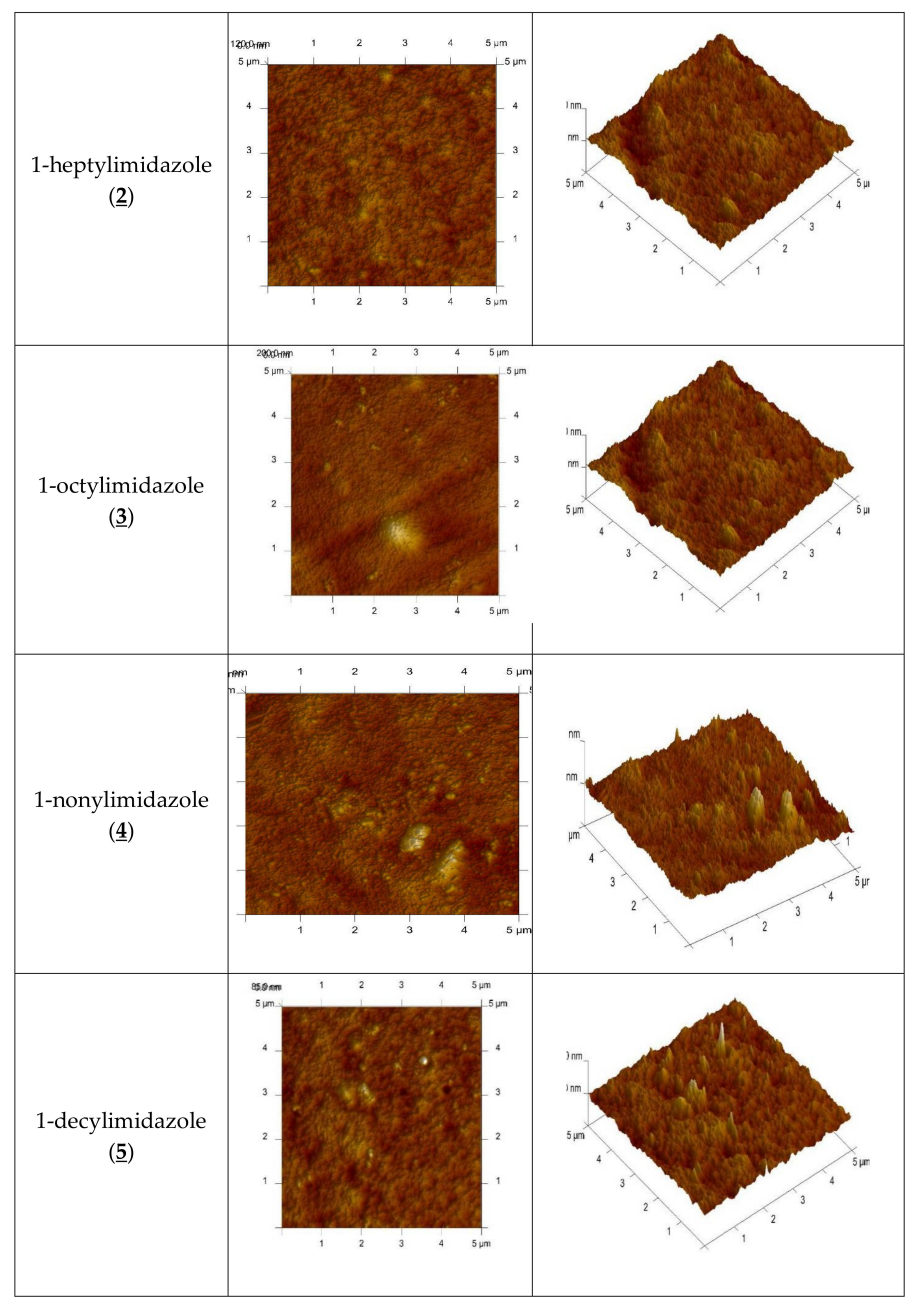
2D- and 3D- view atomic force photomicrographs of PIMs with 1-alkyl-imidazole at a 1 mol/dm^3^ carrier concentration. 2D-view (**a**) and 3D- view (**b**).

An AFM image of the PIM doped with 1-alkyl-imidazole in two- and three-dimensional forms is shown in Figure 4.

In the image of PIM samples (Figure 4) there are clearly visible elongated pores, also called “cavity channels” (darker regions). Such morphology of the membrane surface can be related to the crystallinity of the CTA.

A wide network of pores 5–25 nm in size is likely to be responsible for the improved transport performance of the membranes.

The roughness (*R_q_*) and effective pore sizes of the membrane were calculated using atomic force microscopy (AFM) and they are shown in Table 1 together with the tortuosity of the membrane, determined from the dependence developed by Wolf and Strieder [42]:

τ = 1 − lnε(7)

In the image of PIM samples (Figure 4), there are clearly visible elongated pores, also called “cavity channels” (darker regions). Such morphology of the membrane surface can be related to the crystallinity of the CTA.

A wide network of pores 5–25 nm in size is likely to be responsible for the improved transport performance of the membranes.

The roughness (*R_q_*) and effective pore sizes of the membrane were calculated using atomic force microscopy (AFM) and they are shown in Table 1, together with the tortuosity of the membrane, determined from the dependence developed by Wolf and Strieder [42]: 

The roughness, the effective size of pores and tortuosity of the investigated membranes increase along with an increase in the hydrophobicity of the carrier molecules.

As demonstrated in a number of papers [24,43,44,45,46], the microstructure of the membrane has an impact on the transport process. CTA membranes have porous structures, and the distribution of pores is nearly uniform (porosity of 50%) [41]. The pores in a CTA matrix were filled with a plasticiser (o-NPPE) and the carrier. The carrier crystallises inside the membrane, with the texture of the surface being relatively homogeneous, with varying porosity and roughness. The roughness of a CTA membrane obtained by Tor et al. [46] equalled 14 nm.

The thermal stability of the polymers depends, among other things, on their internal structure, cross-linking and the presence of aromatic rings and degradable functional groups. It was demonstrated in [46] that degradation of a CTA membrane matrix proceeds in two steps: the former in the range of 292–320 °C (main degradation), the latter in the range of 450–476 °C (carbonization). According to other reports, degradation of a CTA membrane is a single-step process occurs at 300 °C [47] or 350 °C [48]. 

**Figure 5:**
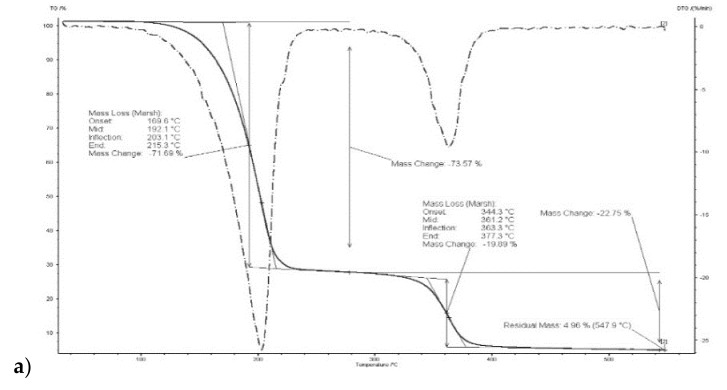

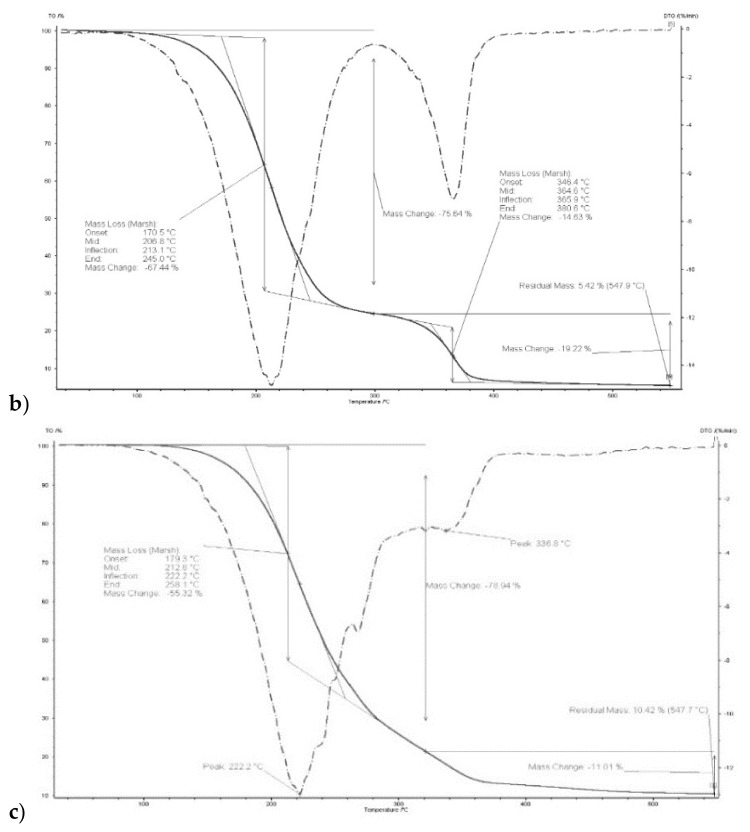
Thermograms of CTA + *o*-NPPE (**a**), CTA + *o*-NPPE + carrier (**5**) (**b**), CTA + *o*-NPPE + carrier (**5**) after process (**c**).

Thermograms of membrane made of CTA – *o*-NPPE with 1-decyl-imidazole (**5**) (Figure 5) indicates that degradation of the membrane proceeds in two steps. For membrane before transport in the first step, at 208.8 °C, the weight loss is 67.44%, in the second at 364.6 °C the weight loss is 14.63%. For this membrane residual mass at 547.9 °C is 5.42%. Moreover, it was reported in [37] that degradation of the CTA—*o*-NPPE membrane with 1-hexylimidazole proceeds in two steps at 211.3 and 360.7 °C. Thus, the results obtained enable the conclusion that polymer inclusion membranes CTA—*o*-NPPE which contain imidazole derivatives show high thermal stability (up to approx. 200 °C).

### 3.2. Transport of Zn(II) and Mn(II) ions across PIMs

Findings on the transport of Zn(II) and Mn(II) ions from equimolar sulphate solutions across PIMs containing 1-alkyl-imidazole (**1**–**5**) as an ion carrier are discussed below. The studies were carried out using two-component solutions containing metal ions, each with a concentration of 0.001 mol/dm^3^. The initial flux values and selectivity coefficients for the transport of metal ions across the PIMs are shown in Table 2.

As indicated by the data shown in Table 2, for all investigated PIMs, the initial flux value for the transport of Zn(II) ions is higher than for Mn(II) ions, and it increases with increasing hydrophobicity of the carrier molecule. This means that in the case of a PIM doped with 1-decyl-imidazole (**5**), the initial flux has the highest value. In the case of a PIM doped with 1-hexylimidazole (**1**), the Zn(II)/Mn(II) selectivity coefficient (*S*) has the highest value. 

**Figure 6:**
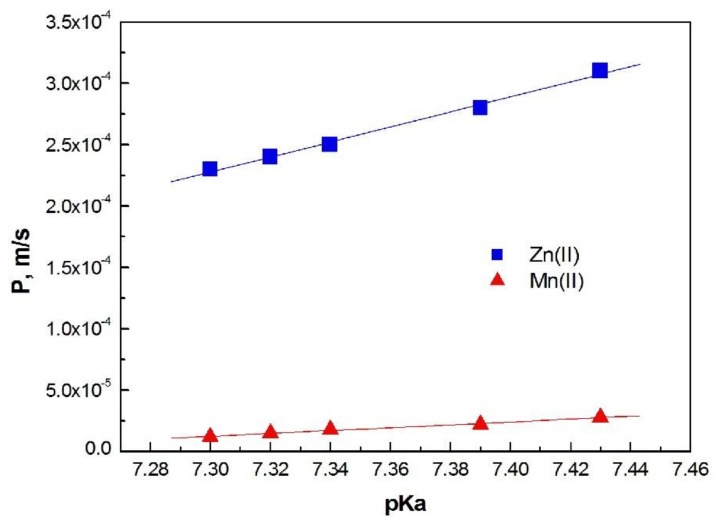
The dependence of the p*K*_a_ 1-alkylimidazole molecules *vs*. permeability coefficients (*P*) for Zn(II) and Mn(II) ions transported across PIMs.

From Equation (3), the permeability coefficient of both ions was calculated for each investigated membrane. Figure 6 shows the dependence of membrane permeability coefficients (*P*) on the dissociation constant (p*K*_a_) of the carrier molecules used in these membranes for Zn(II) and Mn(II) ions.

For both ions, the permeability coefficients increase with an increase in the basicity of carrier molecules (increased p*K*_a_ value); however, for Mn (II) ions this increase is smaller.

The values of permeability coefficient (*P*) increase linearly with increasing basicity of the carrier. A similar linear correlation for 1-alkylimidazoles was obtained in the case of chloride solutions [25]; this is in agreement with literature reports.

A PIM doped with 1-decyl-imidazole (**5**) is the most permeable for Zn(II) and Mn(II) ions. The membrane (**5**) also has the highest effective pore size (Table 2).

### 3.3. Recovery of Metal

**Figure 7:**
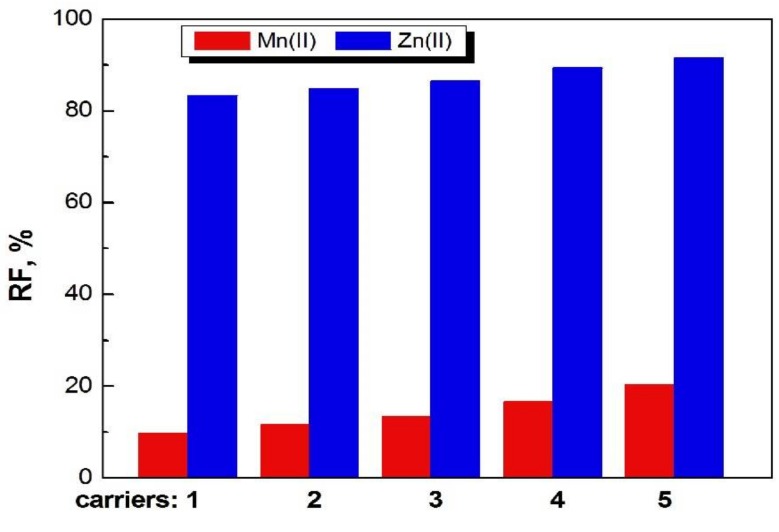
Recovery factors of Mn(II) and Zn(II) ions from feed phase after 24 h in transport across PIM with **1**–**5** compounds at concentration 1.0 mol/dm^3^.

The recovery factors (RF) of Zn(II) and Mn(II) ions as a result of transport by PIMs doped with 1-alkylimidazoles from their equimolar sulphate solutions into deionized water is shown in Figure 7.

The recovery factors for both ions increase in the series: **1**<**2**<**3**<**4**< **5**.

In the case of Zn(II) ions, the recovery factors are much higher than for Mn(II) ions. 

The best result for Zn(II) removal after 24 h was 92.5% for PIMs doped with 1-decyl-imidazole (**5**).

## 4. Conclusions

Zinc(II) ions can be effectively separated from equimolar aqueous solutions of zinc and manganese sulphates by using transport across polymer inclusion membranes doped with 1-alkylimidazoles (alkyl = hexyl, heptyl, octyl, nonyl, decyl). The initial fluxes of Zn(II) and Mn(II) ions increase with an increase in the basicity of carrier (1-alkylimidazole) molecules. The highest initial flux of Zn(II) ions (2.65 µmol/m^2^∙s) was found for PIMs doped with 1-decyl-imidazole, whereas the best Zn(II)/Mn(II) selectivity coefficients equal to 19.7 were found for 1-hexyl-imidazole.

The best result for Zn(II) removal after 24 h was 92.5% for PIMs doped with 1-decyl-imidazole. The microstructure of the membrane has an impact on the transport process. The roughness, the effective size of the pores and the tortuosity of the investigated membranes increase with an increase in the hydrophobicity of carrier molecules, so that the best membranes for Zn(II)-Mn(II) separation are PIMs doped with 1-decyl-imidazole, but for this carrier, the Zn(II)/Mn(II) separation coefficient is the lowest.

It can be assumed that strongly hydrophobic 1-alkylimidazoles (alkyl > decyl) can be more effective in the separation of zinc and manganese ions in membrane techniques.

## Figures and Tables

**Table 1:** AFM characterization parameters for PIM doped with 1-alkyl-imidazole.

Carrier in the CTA-*o*-NPPE Membrane	Effective Pore Size, µm	Tortuosity	Roughness (*R_q_*), nm
1-hexyl-imidazole (1)	0.050 ± 0.002	2.32	5.90 ± 0.05
1-heptyl-imidazole (2)	0.053 ± 0.002	2.35	6.10 ± 0.05
1-octyl-imidazole (3)	0.054 ± 0.002	2.38	6.50 ± 0.05
1-nonyl-imidazole (4)	0.055 ± 0.002	2.64	6.70 ± 0.05
1-decyl-midazole (5)	0.057 ± 0.002	2.81	7.20 ± 0.05

**Table 2:** Initial fluxes, selectivity order and selectivity coefficients for competitive transport of Zn(II) and Mn(II) ions across PIM doped with 1-alkylimidazole; membrane: 2.6 cm^3^ *o*-NPPE /1g CTA and 1.0 mol/dm^3^ carriers calculated on plasticizer, pH of the feed phase—5.6, receiving phase—deionized water.

Carrier	Metal Ions	J, μmol/m^2^⋅s	*S*_Zn(II)/Mn(II)_
**1**	Zn(II)	1.97	Zn(II) > Mn(II) 19.7
	Mn(II)	0.10	
**2**	Zn(II)	2.04	Zn(II) > Mn(II) 15.7
	Mn(II)	0.13	
**3**	Zn(II)	2.13	Zn(II) > Mn(II) 14.2
	Mn(II)	0.15	
**4**	Zn(II)	2.43	Zn(II) > Mn(II) 12.8
	Mn(II)	0.19	
**5**	Zn(II)	2.65	Zn(II) > Mn(II) 11.0
	Mn(II)	0.24	
